# An Episode of Psychosis After Nitrous Oxide Abuse During a Pandemic: A Case Report

**DOI:** 10.7759/cureus.60634

**Published:** 2024-05-19

**Authors:** Shaeraine Raaj, Mian Saad Ahmed, Emma Warren, Richard Farrelly

**Affiliations:** 1 Psychiatry, Connolly Hospital Blanchardstown, Dublin, IRL; 2 Psychiatry, Phoenix Care Centre, Dublin, IRL; 3 Medicine, Trinity College Dublin, Dublin, IRL; 4 Psychiatry, Tallaght University Hospital, Dublin, IRL

**Keywords:** multi-disciplinary team approach, inhalation, nitrous oxide abuse, covid-19 pandemic, first episode psychosis

## Abstract

The abuse of inhalants like nitrous oxide (N_2_O), readily available worldwide, has remained a prominent public health problem during the last few decades. Literature reveals increased use during the previous pandemic, particularly regarding recreational use. There is limited evidence-based data available to relate the abuse of N_2_O with psychosis. Therefore, this case report of a 22-year-old adult with no previous psychiatry history, reportedly abusing 75-100 canisters of N_2_O per day during the last pandemic COVID-19 lockdown, highlights the relationship between (N_2_O) abuse and the symptoms evolved including delusions, auditory hallucinations, and disorganized cognition. All the laboratory findings and results from imaging modalities were inconsistent for any organic cause of the symptoms. The case then underwent treatment with antipsychotic medications and a multidisciplinary model, which improved the symptoms gradually. The case, in particular, discusses N_2_O abuse, which is widespread in European Union countries, including the UK and the Republic of Ireland, and its chronic use puts one at a higher risk of developing psychosis, personality changes, affective lability, anxiety, depression, cognitive impairment, and myeloneuropathy. The sale of N_2_O for its psychoactive properties is prohibited in many countries, including the Republic of Ireland, as per legislation. However, N_2_O is not a controlled drug, meaning it is not a crime to possess N_2_O. This case report manifests the psychopathy caused by abuse of N_2_O, which would further attract specialists in the field to conduct epidemiological studies for prevention at the primary level.

## Introduction

Historically, nitrous oxide (N_2_O) is also known as "laughing gas" and is clinically used in medical and dental settings [1-4]. N_2_O was discovered in 1775 by Joseph Priestley, who also discovered oxygen in 1774 [5]. In the early 1800s, N_2_O was classified as a safe anesthetic medication mixed with 30% oxygen and recognized for its anti-anxiety effect with limited use nowadays as well [1,3]. The short half-life of N_2_O is a significant advantage during its use in childbirth, dental surgery, and emergency settings [6]. As a result of its chemically inert and bacteriostatic properties, N_2_O does not leave any taste or odor and is widely used in whipped cream canisters or cartridges [3,4]. Additionally, the same gas is known to be a “laughing gas” or “Hippy Crack” for its euphoric and hallucinogenic effects that disappear in minutes [2-4]. Due to its therapeutic effect, it is widely available legally in many countries but has also been reported to be associated with some 36 deaths based on excessive use between 2001 and 2016 in the United Kingdom, a country where this gas is the second most common recreational substance [7,8].

Since the beginning of the recent COVID-19 pandemic (also known as coronavirus), an increased misuse of N_2_O as a recreational drug has been noted in the Republic of Ireland, the United Kingdom, and the Netherlands [9-12]. Recent research reports that as a result of travel restrictions with international shipments during the lockdown (March 2020 to mid-2021), there have been challenges in transporting different illicit substances, including cannabis, opioids, and cocaine, subsequently leading young people to use this euphoric gas as a substitute [9,7,11-12]. In Ireland, there has been a spike in the number of N_2_O canisters discovered in parks, streets, and inner Dublin City since April 2020 [9]. The Irish Times in September 2021 reported, "Revenue made no seizures of nitrous-oxide between 2015 and 2019, but in the first half of 2020, seized two shipments, totaling 14,400 canisters, which were believed to be destined for the illicit market" [9]. The use of N_2_O is regulated in Ireland by the Criminal Justice (Psychoactive Substances) Act 2010, and the sale, importation, exportation, and advertisement of N_2_O are prohibited [7]. However, N_2_O is not a controlled drug in the Republic of Ireland; therefore, it is not a crime to possess N_2_O [7]. The giant online retailer Amazon ceased selling N_2_O in Ireland in 2021 [7]. However, there are other online resources, including catering companies shipping N_2_O into Ireland.

When inhaled, N_2_O acts as an N-methyl-D-aspartate (NMDA) antagonist similar to the neurotoxic effect of ketamine, resulting in a decrease in excitatory neurotransmission through the CNS via non-competitive glutamate inhibition [3-4]. Additionally, N_2_O acts as a partial mu, kappa, delta, and opioid receptor agonist, modulating dopamine activity within the nucleus accumbens (reward pathway) and noradrenergic pathway via midbrain peptide release [4]. Inhaling N_2_O leads to euphoria with psychedelic effects, including feelings of dissociation and mild changes in perception of the body image [3-4]. Recent studies have reported vitamin B12 (cyanocobalamin) deficiency in people abusing N_2_O, resulting in psychiatric complications such as psychosis [2,4,13-14]. Einsiedler and colleagues reported on a case series with five patients presenting with neurological complications following prolonged use of N_2_O [11]. Among five patients, 4/5 patients presented with subacute combined spinal cord degeneration, and 1/5 patients presented with inflammatory demyelinating polyneuropathy.

The toxicity effect of N_2_O has only been reported in the last several years. The reported cases of NO toxicity primarily reported neurological and psychiatric presentations. The case report aims to improve clinicians' awareness of N_2_O and discuss a clinical case of psychosis in a young man who started abusing N_2_O "canisters" during the COVID-19 pandemic lockdown.

## Case presentation

A 22-year-old single working man was living with his mother and siblings in the family home. His brother accompanied him to an Emergency Department in a General Hospital in the Republic of Ireland following a six-month history of third-person auditory hallucinations, delusions of persecution, somatic passivity, and personality changes.

The patient’s past medical and psychiatric history was unremarkable. There were no psychiatric disorders in the family history. He has a history of using cannabis since the age of 19. He was introduced to cannabis by acquaintances, and he reported that his cannabis intake gradually progressed from one joint a day to five joints a day.

The patient stopped using cannabis and started using N_2_O during the COVID-19 pandemic lockdown due to the challenge of purchasing cannabis. He reports inhaling N_2_O to experience rapid euphoria and described inhaling approximately 75-100 canisters (8 to 20 grams) of N_2_O per day for nearly six to nine months. He reported that N_2_O canisters were easily purchased on the internet from an online commercial retailer that ships directly to the user’s doorstep. He said that the wholesale provider sells canisters of varying sizes and encourages bulk purchases to avail for a discounted price. The patient was unaware of the psychiatric risk factors involved with inhaling N_2_O. He said the canisters were not sold for inhalation purposes but for balloon inflation. He reported having last used N_2_O three days before he presented to the emergency department.

In the six months leading to his presentation to the hospital, he had become increasingly distressed and preoccupied with third-person auditory hallucinations, persecutory delusions, and delusions of control. He believed he was under constant surveillance by his neighbors and the Gardai (police), who were looking to implicate him in a drug ring. He also thought that the Garda had implanted a microchip in his right arm and was transmitting his thoughts. The patient was convinced that the Garda had placed cameras in his home and tracked his phone and he was also troubled by thoughts of worthlessness which he believed were inserted into his mind by his neighbor.

On evaluation, his vital signs were stable. He showed no sign of head trauma on examination, he had a Glasgow Coma Scale (GCS) of 15, and his pupils were equal and reactive to light. A thyroid, respiratory, cardiac, and abdominal examination was unremarkable. His neurological examination results were normal, and he had no acute neurological symptoms.

Laboratory investigations reported full blood count, glucose, urea and electrolytes, thyroid function test, liver function test, and HbA1c within normal limits. The vitamin B12 immunoassay result was 402 ng/L (normal range: 187-883 ng/L). Urine toxicology results were negative. A computed tomography scan (CT) of the brain was completed to rule out any organic etiology. There was no evidence of intracranial abnormalities as per the CT brain scan, and the brain parenchyma and ventricular system were normal.

The patient was admitted to the psychiatry unit as a voluntary patient. He presented as well-groomed and neat. He was cooperative but guarded, suspicious, and demonstrated intermittent eye contact. He had a blunted affect with a euthymic mood and smiled incongruently during the review. His speech was coherent with a high-pitched tone. He described a systematized persecutory delusion and reported third-person auditory hallucination, including running commentary. His thought form was linear, and he did not express suicidal or homicidal ideation. He had no insight but agreed to remain in the hospital as a voluntary patient. He was easily distracted and was paranoid about his safety. The initial differential diagnoses were substance-induced psychotic disorder, paranoid schizophrenia, or an organic psychosis.

The patient presented with limited insight into his mental state during his inpatient stay. He did not believe he was unwell and was perplexed by his admission. Oral olanzapine 10 mg was prescribed once at night to treat psychotic symptoms. He continued to describe the third person's auditory hallucinations, running commentary hallucinations, and paranoid delusions. The dose of olanzapine was increased to 15 mg nocte and, subsequently, 20 mg daily (once at night) for psychotic symptoms. He gradually became less preoccupied with his delusional belief system, less suspicious, and less guarded. Over time, he maintained stability in his mental state wherein hallucination and delusion stopped with antipsychotic medications. The patient was discharged from the inpatient setting after four weeks of inpatient treatment on 20 mg of olanzapine. While planning the discharge, the patient reported that he was initially unaware of the adverse effects of using N_2_O and said that he is eager to remain abstinent from using N_2_O. According to the transtheoretical model stages of change, the patient presented in the preparation stage of changes and expressed a desire and motivation to engage with the local addiction services. He was followed up by the home-based treating team (HBTT) at the community mental health outpatient setting, wherein he was linked to the local addiction service. Day program intervention and cognitive behavioral therapy were delivered in the community setting. He was engaged with a psychiatrist, community nurse, psychologist, mental health social worker, and occupational therapist to promote daily living and routine. The HBTT provided close follow-up (weekly) following discharge from the acute psychiatric unit. The HBTT provided weekend reviews and management to reduce the risk of relapse and breakthrough systems. Additionally, the patient was offered depot which he refused so he was kept on 20 mg of olanzapine for a while which later was tapered.

## Discussion

**Figure 1:**
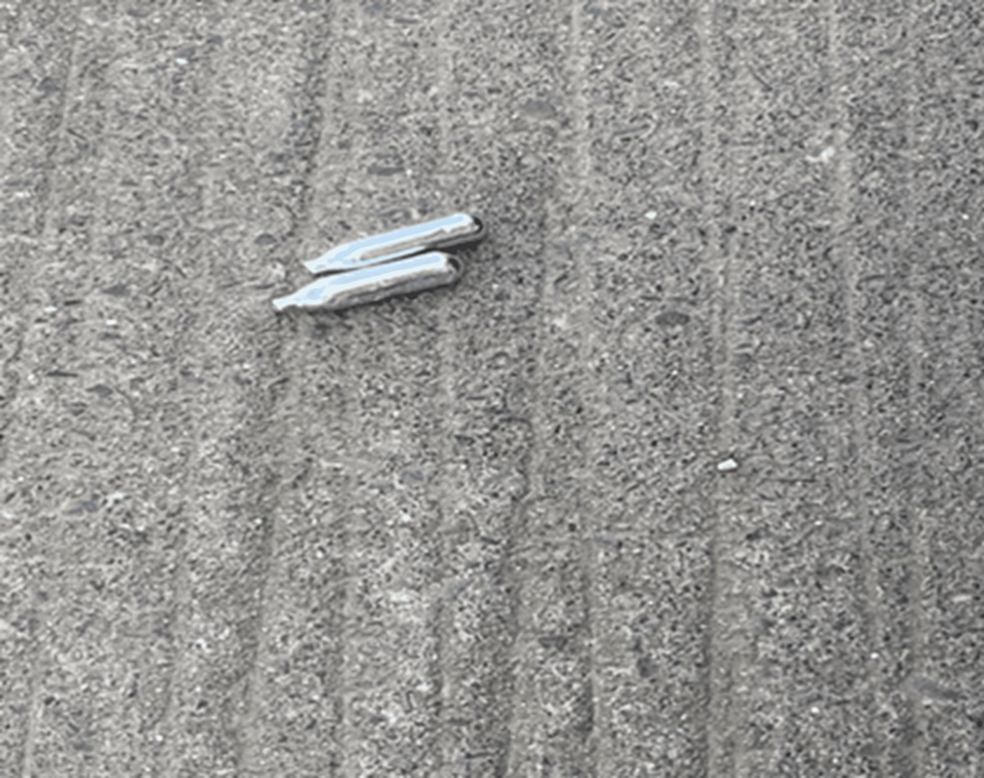
Canister of nitrous oxide found on the street in Dublin, Ireland

This case reports psychotic symptoms in a person as a result of N_2_O abuse. The patient stopped using cannabis, and the onset of the psychotic symptoms was preceded by an increase in consumption of inhaled N_2_O via canister as shown in Figure 1. Gas abuse has been increasing during the COVID-19 pandemic lockdown and is known to cause cobalamin deficiency due to its mechanism of action [10-12].

The literature describes how N_2_O inactivates vitamin B12 via irreversible oxidation of the cobalt core from the monovalent to the trivalent state [13]. The exact pathophysiology behind the development of psychiatric comorbidities in N_2_O users remains unknown. A study published in 2011 in Australia provided a hypothesis about increased nitric oxide production via activation of the pre-synaptic nitric oxide synthase enzyme [15]. Nitric oxide reacts with superoxide free radicals and forms peroxynitrite, a potent oxidant and neurotoxin. N_2_O acts as a non-competitive NMDA antagonist, leading to its psychogenic properties [3-4].

The gold standard investigation is to measure the vitamin B12 level to assess cobalamin deficiency; however, it might remain normal in a few cases [3-4,12-13]. The management of N_2_O-induced psychosis includes abstinence from N_2_O use and short-term oral or intramuscular cobalamin supplementation; however, as this case did not report any deficiency of vitamin B12, so medication was offered for the same purpose [13]. Literature has covered few studies wherein after the use of N_2_O, psychotic symptoms were developed but levels of vitamin B12 were normal, which further needs to be explored for its effect [3-4,12-13]. Additionally, the risk associated with not commencing vitamin B12 supplementation increases the risk of homocysteine and methylmalonic acid production, which is attributed to demyelination of the spinal cord [12]. Similarly, as the particular case had normal vitamin B12 levels, investigations were not performed for homocysteine and methylmalonic acid.

In the DSM-V, the distinction between substance-induced psychosis and schizophrenia is based on the persistence of psychosis beyond one month after the last exposure to the implicated substance [16]. The one-month criteria is arbitrary but based on a comprehensive review of relevant data. The transition from substance-induced psychosis to schizophrenia is approximately 25% [16-17]. The type of substance is the primary predictor of the transition from substance-induced psychosis to schizophrenia, with the highest rates associated with cannabis [17-18]. Our patient has been abstinent from cannabis and presented with a 16-18 months history of abusing N_2_O and had last used N_2_O three days before attending the hospital. Similarly, his treatment with antipsychotics in line with clinical indications went well and his psychotic symptoms were improved.

The evidence of a global increase in N_2_O abuse, particularly after the COVID-19 pandemic was covered and explained in this case report. There is limited awareness about the risks associated with prolonged N_2_O use and diagnostic criteria for dependence. Therefore, we advocate a combined effort of legislation in restricting the availability of N_2_O and better educational campaigns via mental health awareness campaigns (including N_2_O information in existing prevention strategies). There is a need for further training for hospital doctors to measure vitamin B12 levels when treating young people with neurological and psychiatric symptoms, and treat as required. Input by legislative institutions to target abuse of this gas is another preventive step that is direly needed. Epidemiological studies are required to better understand causative factors for the increased prevalence of N_2_O use in the Republic of Ireland. Research can help highlight the adverse effects of N_2_O by increasing awareness of N_2_O and early prevention and management among a wide range of stakeholders.

## Conclusions

The case report concludes the rare presentation of evidence of psychotic symptoms in a case of N_2_O abuse that was well treated and managed via the use of antipsychotics and multi-disciplinary psychiatric input. Furthermore, the case report will highlight different preventive steps to be taken by relevant organizations to address its abuse at a primary level, achieving high standards of prevention.
